# Comparative analysis of imaging diagnostic models for tubular basophilia and mineralization of kidney

**DOI:** 10.1186/s42826-022-00139-y

**Published:** 2022-09-15

**Authors:** Jong Su Byun, Ji Hyun Lee, Jin Seok Kang, Beom Seok Han

**Affiliations:** 1 Department of Pharmaceutical Engineering, College of Natural Science, Hoseo University, Beabang-eup Hoseo-ro 79-20, Asan-si, 31499 Chungcheongnam-do Korea; 2 College of Software Convergence, Kwangwoon University, Kwangwoon-ro 20, Nowon-gu, Seoul-si, 01897 Korea; 3 Department of Biomedical Laboratory Science, Namseoul University, 91 Daehak-ro, Seonghwan-eup, Seobuk-gu, Cheonan City, 31020 Korea

**Keywords:** Artificial intelligence, Diagnosis, Classification models, YOLOv4, Tubular basophilia, Mineralization

## Abstract

**Background:**

Now that it is possible to efficiently classify and save tissue images of laboratory animals using whole-slide imaging, many diagnostic models are being developed through transfer learning with Convolutional Neural Network (CNN). In this study, transfer learning was performed to gain toxicopathological knowledge using CNN models such as InceptionV3 and Xception. For the classification of tubular basophilia and mineralization, two representative background lesions that commonly occur in toxicological studies, accuracies of diagnosis were compared using MobileNetV2, Xception and InceptionV3. For the simultaneous detection of the two lesions, the accuracy was analysed using You Only Look Once version 4 (YOLOv4).

**Results:**

The accuracy of the classification models was as follows: MobileNetV2 (epoch 50, accuracy: 98.57%) > Xception (epoch 70, accuracy: 97.47%) > InceptionV3 (epoch 70, accuracy: 89.62%). In the case of object detection, the accuracy of YOLOv4 was 98.62% at epoch 3000.

**Conclusions:**

Among the classification models, MobileNetV2 had the best accuracy despite applying a lower epoch than InceptionV3 and Xception. The object detection model, YOLOv4, accurately and simultaneously diagnosed tubular basophilia and mineralization, with an accuracy of 98.62% at epoch 3000.

## Background

Artificial intelligence (AI) is a field of computer science that is defined as enabling computers to mimic human intellectual behaviour. AI is being applied to various fields in the twenty-first century owing to its high accuracy and very fast task processing through massive data learning [1]. Medical and pathological image analysis technology using AI is positioned to lead the development of the field of AI-based imaging [2]. In particular, imaging area of AI is also currently being actively applied and studied in the field of toxicopathology.

In this field, the term “classification” refers to classification of an object in an image as an input. For example, when an image of renal mineralization is presented as an input, it is classified as “This photo is an image showing mineralization”. In this example, mineralization classified by the computer is named “label” or “class”. Representative classification models include AlexNet [3], VGGNet [4], Inception [5], and MobileNet [6], all of which have a CNN structure. Lung cancer has been classified with 97% accuracy using the InceptionV3 model, and when the CNN subtype neural network was trained on images from lung adenocarcinoma patients combined with mutation profiles, it was able to predict the presence of mutations in specific genes [7]. Furthermore, models based on CNN, such as VGGNet, ResNet, and AlexNet, have been proposed and their accuracy has been tested [8].

The term “localization” refers to the location of an object in an image using a bounding box as well as a simple classification feature. “Object detection” suggests that classification and localization are performed simultaneously on multiple objects. For example, using an object detection model, it is possible to classify and localize different lesions in the rat kidney. The YOLO model is a representative object detection model [9].

‘YOLO’ stands for ‘You only look once’ and is a method that improves accuracy by applying the grid cell and Bounding Box method, which indicate specific image, to overcome the shortcomings of the existing sliding window method [9, 10]. In addition, it boasts a fast detection speed and high accuracy, and is currently the most widely used object detection model [9, 10]. The network structure of the YOLO model is based on the GoogleNet model [11] and consists of 24 convolutional layers and 2 fully connected layers. YOLOv4 is more accurate and faster than YOLOv3 owing to a 10% improvement in mean Average Precision (mAP) and a 12% increase in the number of frames per second. In addition, the YOLOv4 model is equipped with an inherent function called “Mosaic augmentation”, and which is characterized by easy statistical calculation of batch normalization using a method to predict specific object by merging four images into one [12].

Transfer learning is used to perform additional image learning using previously trained CNN models (classification models, object detection models, etc.). In general, the learning speed and final algorithm accuracy are better if transfer learning is performed to learn knowledge in a specific field from an existing AI model rather than by developing a new algorithm and AI model in a specific field [13].

Toxicopathology is a field to morphologically evaluate the efficacy and safety of test substances. It plays an important role in diagnosing diseases and identifying causes, and in providing rational grounds and directions for treatment development [14]. In general, toxicopathologists diagnose and evaluate lesions in tissues of laboratory animals on glass slides. Whole Slide Images (WSIs) are digitized image of classical slide glass samples using a virtual slide scanner. This WSI technique has established itself as a means to perform pathological evaluation [15], and is being converted to virtual microscopy based on WSI in the field of pathology research and education [16].

Tubular basophilia and mineralization are two commonly spontaneous lesions in the kidney of rodents [17]. Tubular basophilia is characterized by tubular epithelial cells with a basophilic cytoplasm; the cells are slightly enlarged, which is used as a diagnostic feature. It occurs in an early stage of chronic progressive nephropathy (CPN) and is observed with an increasing incidence during rodent aging [17]. Mineralization, commonly known as calcification, occurs frequently at the cortex–medullary junction in rodents and is characterized by the replacement of tubular cytoplasm with deposits due to tubular degeneration [18]. These lesions are major component of CPN, which sometimes misdiagnosed by senior pathologist and could develope renal tumor. The reason for the use of these two lesions for transfer learning is that they are very common naturally occurring background lesions in rodent kidneys and have distinct characteristics that even junior pathologists can easily diagnose, so each model can be easily trained. In this study, the accuracy of classification of tubular basophilia and mineralization was compared using the AI classification models InceptionV3, Xception, and MobileNetV2. In addition, the accuracy of simultaneous detection of the two lesions was calculated using YOLOv4.

## Results

### Accuracy evaluation of classification models

**Fig. 1:**
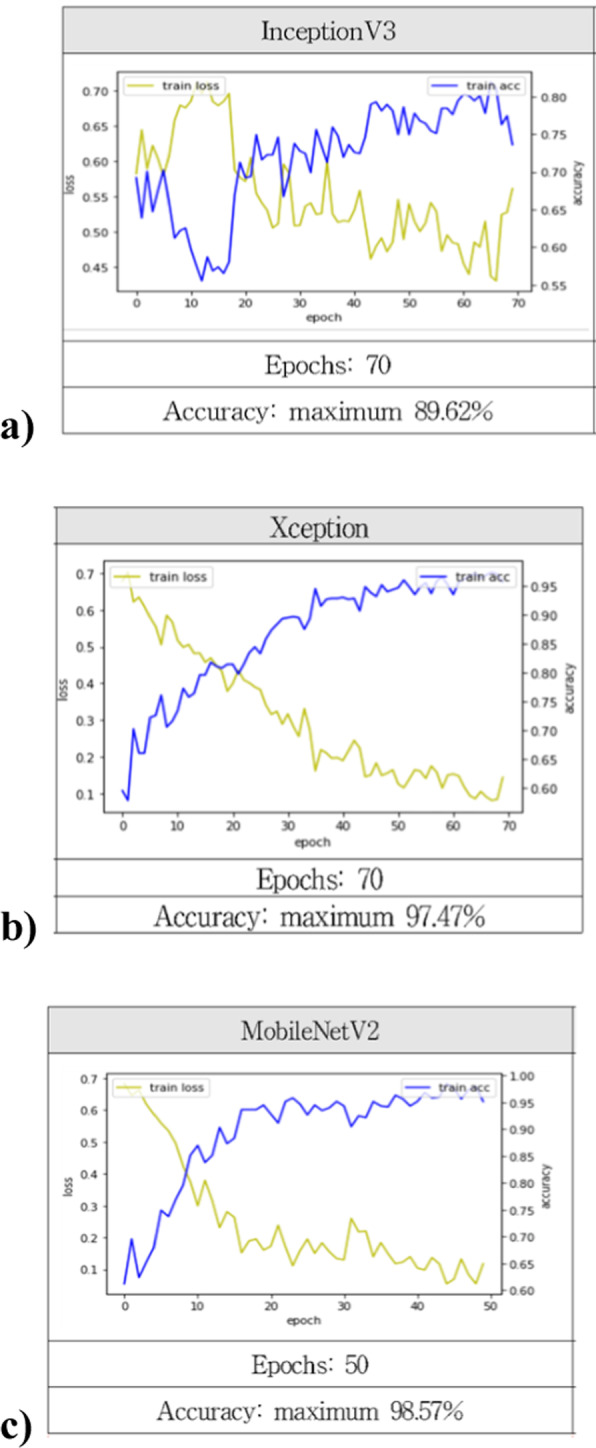
Accuracy evaluation of classification models. **a** InceptionV3. by The accuracy was 89.62% in epoch 70. **b** Xception. The accuracy was 97.47% in epoch 70. **c** MobileNetV2. The accuracy was 98.57% in epoch 50. The accuracy of each model was calculated after transfer learning of toxicological knowledge

The maximum accuracy was 89.62% in InceptionV3 (Fig. 1a), 97.47% in Xception (Fig. 1b), and 98.57% in MobileNetV2 (Fig. 1c). Therefore, the accuracy of the classification models was as follows: MobileNetV2 > Xception > InceptionV3.

### Classification of tubular basophilia and mineralization using MobileNetV2

**Fig. 2:**
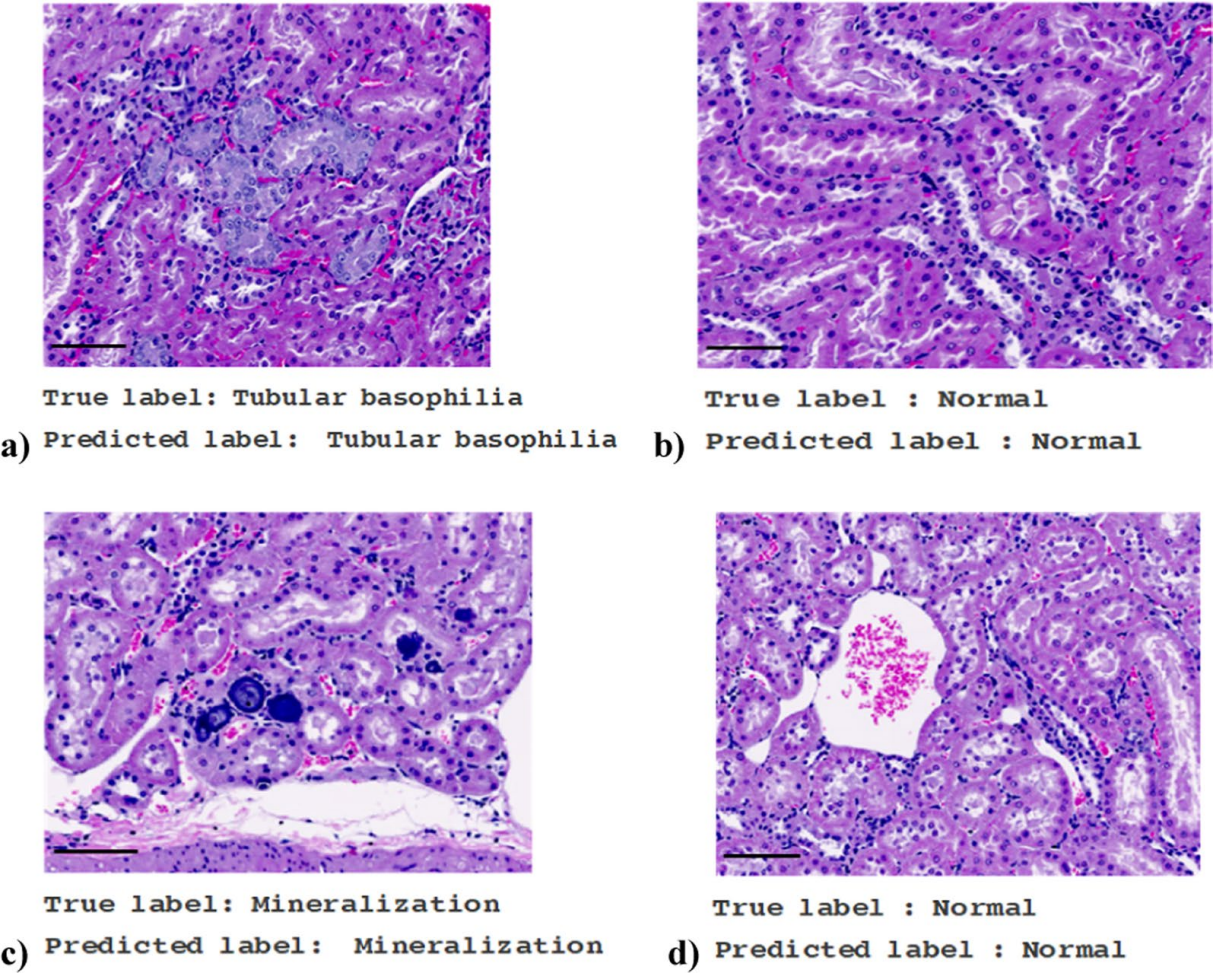
Diagnosis by MobileNetV2. Transfer-learned MobileNetV2 diagnosed the test sample (WSI, scale bar = 200 µm, original magnification × 200). **a** Diagnosis of tubular basophilia. Tubular basophilia was accurately classified and diagnosed. **b** Diagnosis of normal kidney. Normal tissue but not the lesion site was diagnosed as normal. **c** Diagnosis of mineralization. Mineralization was accurately classified and diagnosed. **d** Diagnosis of normal kidney. Normal tissue but not the lesion site was diagnosed as normal

MobileNetV2 was trained through image training in 50 epochs. As a result, it was possible to classify and diagnose tubular basophilia (Fig. 2a) and mineralization (Fig. 2c). In addition, non-lesion sites were clearly diagnosed as normal (Fig. 2b, d).

### Accuracy evaluation of object detection models

**Fig. 3:**
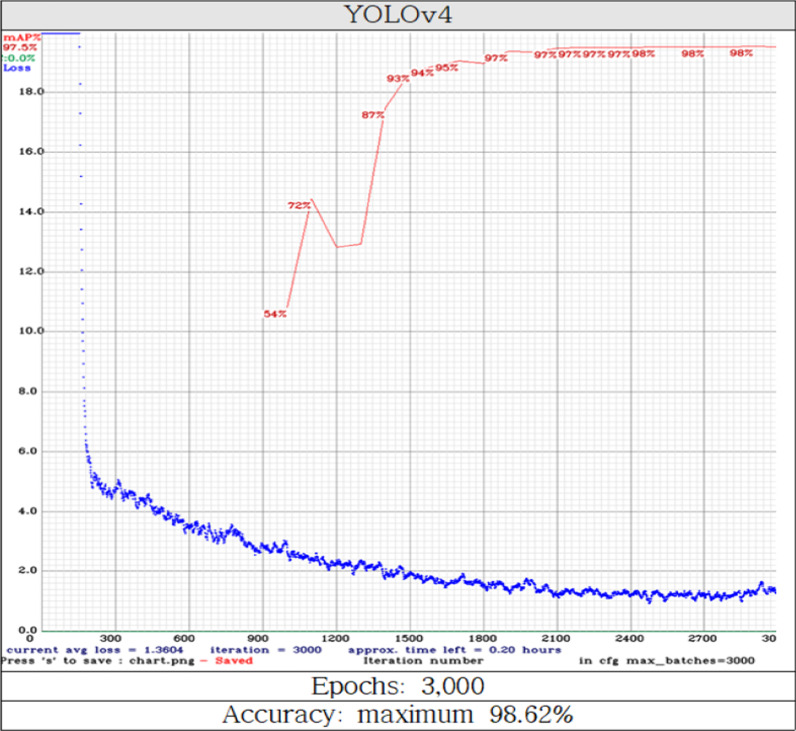
Accuracy evaluation of the object detection model YOLOv4. The *x*-axis indicates epochs and the *y*-axis indicates loss range. Red text, mAP; blue text, the loss value. Each time the epochs (training cycles) increase, the accuracy is updated and calculated, and the loss value is calculated at the same time. Epochs and loss values are inversely proportional. Below 1,000 epochs, the accuracy was not calculated. This is the mechanism of the object detection model, which means that at least a certain number of epochs must be applied before accuracy is calculated

**Fig. 4:**
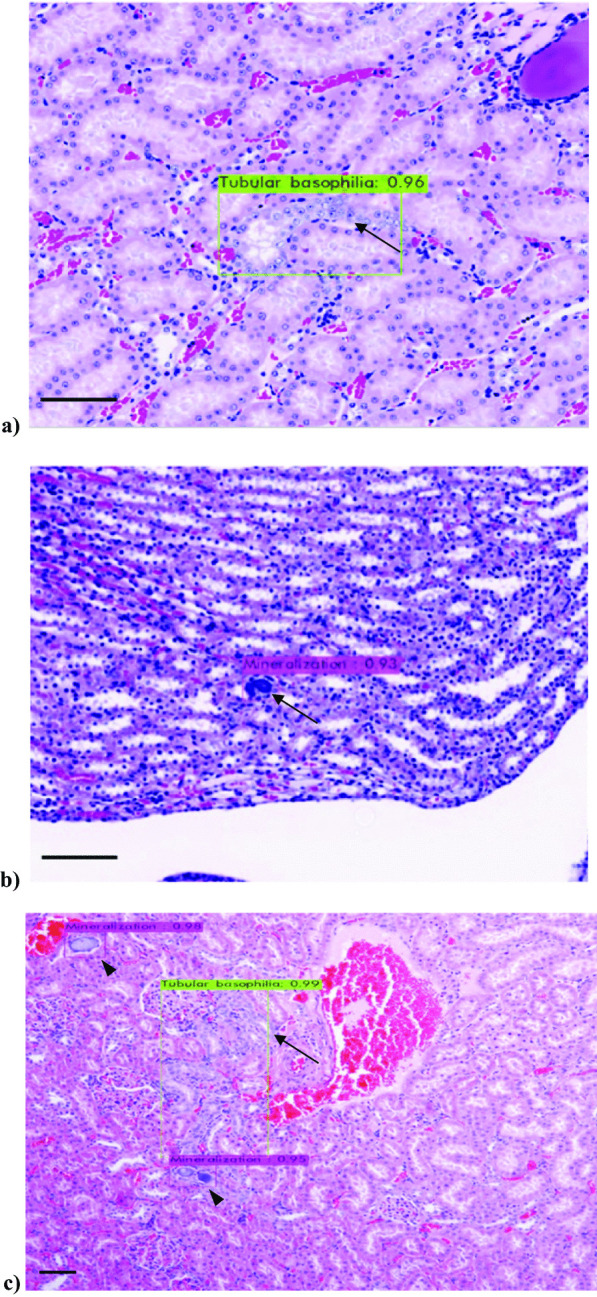
Diagnosis by YOLOv4. **a** Recognition of tubular basophilia (arrow) in WSI (Scale bar = 200 µm, original magnification × 200). YOLOv4 accurately diagnosed renal WSI, to which the brightness control function, which is an inherent function of WSI, was applied. The location of tubular basophilia was detected using the bounding box, the output of YOLOv4. **b** Recognition of mineralization (arrow) in WSI (Scale bar = 200 µm, original magnification × 200). The location of mineralization was detected using the bounding box, the output of YOLOv4. **c** Simultaneous recognition of tubular basophilia (arrows) and mineralization (arrowheads) in WSI (Scale bar = 200 µm, original magnification × 100). Tubular basophilia and mineralization were simultaneously detected using the bounding box, the output of YOLOv4

The maximum accuracy of YOLOv4 subjected to deep learning was 98.62% (Fig. 3). The mAP of YOLOv4 was 0.9862. (mineralization, 0.9904; tubular basophilia, 0.9820). YOLOv4 that deep learned in epoch 3,000 diagnosed tubular basophilia and mineralization (Fig. 4a, b). Tubular basophilia and mineralization were simultaneously detected when they were present in adjacent regions in the same image (Fig. 4c).

## Discussion

This study demonstrated that, among the classification models for which toxicopathological knowledge transfer learning was performed, MobileNetV2 had better accuracy than InceptionV3 and Xception. In addition, transfer learning and deep learning were performed using YOLOv4, and the maximum accuracy in epoch 3,000 was as 98.62%.

First, before proceeding with deep learning, the YOLO model (an object detection model) was trained with the WSI to efficiently and accurately identify mineralization and tubular basophilia lesion images. In classification models, when an image is given as an input, one value (result of input) is produced. The one value indicates the level of the highest category by outputting the probability distribution of mineralization and tubular basophilia images. However, the YOLO model, object detection model, indicates four values of the lesion location. Therefore, we thought that this object detection model would be more suitable for the detection of the exact positions of tubular basophilia and mineralization than a classification model, and we finally selected the YOLO model and proceeded with deep learning (epoch 3,000). We selected YOLOv4 rather than YOLOv3 as the former but not the latter has an inherent function for data augmentation. In addition, in terms of overall accuracy, YOLOv4 is an upgraded model that complements YOLOv3. The number of lesion image data was increased to about 1,500 image data using mosaic augmentation technique.

Xception had higher accuracy in epochs 70 (97.47%) than InceptionV3 (89.62%). Xception is a model devised by having perform a depthwise separable convolution operation based on InceptionV3. In a recent study that compared the accuracy of Xception and InceptionV3, which both performed transfer learning, Xception had higher accuracy than InceptionV3, although the learning time was longer in Xception than InceptionV3 in the same epoch [19]. On the other hand, in the case of MobileNetV2, the highest accuracy (98.57%) was found at epoch 50 lower than epoch 70 for both models (InceptionV3 and Xception). Similar to Xception, MobileNetV2 uses a depthwise separable convolution operation that increases the efficiency of the convolution operation; furthermore, it uses a linear bottleneck that can reduce information loss in a nonlinear activation function, and an inverted residual block that increases the internal channel. Since this model can make predictions lighter by using depthwise separable convolution operation, it is estimated that the accuracy is higher than those of the two models even at low epochs [6].

The maximum accuracy of YOLOv4 in epoch 3,000 was 98.62%. Learning was performed with a higher epoch than that of the classification models. There are clear algorithmic differences (input, output, etc.) between the classification models and object detection models, and the accuracy of YOLOv4 was not calculated in epochs less than 1,020 (Fig. 3). In YOLOv4, The accuracy slope increased rapidly from epoch 1,300 or higher, and from epoch 1,860 or higher, a rather shallow slope with an accuracy of 97% or more and a low loss value (< 2.0) were calculated (Fig. 3).

When performing transfer learning of the YOLO model, "underfitting" occurred as a result of training using fewer than 300 lesion images. In general, in an object detection model, image data ranging from a minimum of several thousand to a maximum of tens of thousands are required to detect an object with clear image characteristics. However, since the data given to the YOLO model contained unfortunately fewer than 300 images because of a limited slide sample, "underfitting" occurred because it was difficult to train the image features when training only with basic images (jpg. and basic WSI images). As a solution, YOLOv4 was trained by increasing a total of 288 basic images (containing tubular basophilia and mineralization) to 1,436 by performing data augmentation, an inherent function in YOLOv4. The augmented lesion data were sufficient to train the YOLOv4 model. However, in order to develop a perfect toxicopathology diagnostic model, it will require to have at least several tens of thousands of images to learn various cells that exist in normal tissues and organs for each target organ. Furthermore, if testing is performed on slides with artefacts similar to tubular basophilia or mineralization (transfer learning for target lesions), it could result in misdiagnosis or very low accuracy. As a solution, it is necessary to learn a lot of slide cases in which various artefacts exist to clearly distinguish between normal tissues and lesions. From our experience of performing pathology peer review of Korea National Toxicology Program (KNTP) toxicity study project of Ministry of Food and Drug Safety (MFDS), when examining a pathological slide, at least 1,000 pieces of reading per substance should be inspected.

Furthermore, if deep learning is performed on lesions in various tissues according to diagnostic characteristics, an AI model can quickly pre-screen to examine slides containing or removed lesions targeted by the pathologist. If so, toxicopathologists will be able to finally confirm the results of pre-screened lesions, which will significantly save the lesion reading time and manpower.

The WSI program did not have the image segmentation function and the function to save the images as jpg. files, so I used the Photoscape X program to divide it. In other words, if the compatibility between the virtual environment called GoogleColab and the WSI program is facilitated, AI diagnosis of laboratory animals will become easier.

In this paper, each model was transferred to tubular basophilia and mineralization, which can be easily diagnosed by junior pathologists and commonly occur spontaneous renal lesions. Furthermore, additional learning of lesions such as hyaline cast and renal inflammatory cell infiltration will play a major role in diagnosing CPN, which well-known for complicated spontaneous lesion of kidney and misdiagnosed by senior pathologist.

## Conclusions

Among classification models, as a result of performing transfer learning of toxicopathological knowledge, MobileNetV2 had excellent accuracy despite applying a lower epoch than InceptionV3 and Xception. The object detection model YOLOv4 had an accuracy of 98.62% at epoch 3,000 and accurately and quickly diagnosed tubular basophilia and mineralization in the rat kidney using a bounding box.

## Methods

### Experimental environment

In this study, 288 images of kidney lesions (tubular basophilia and mineralization) in rats (Sprague–Dawley and Fischer 344 rat) used in the KNTP project of the MFDS, which was performed from 2018 to 2020 (13-week repeated dose toxicity test, 3 cases; mineralization: 147 images, tubular basophilia: 141 images, file name extension:.jpg), and 20 WSI slides with kidney lesions (file name extension:.czi) were used to train and validate classification and object detection models. Of the 288 images, 241 were used as training data, and 47 were used as valid data and test data. In addition, 10 of the 20 WSI slides were used as training data and 10 were used as valid data and test data. Google Colab Pro was used as the development environment, and Tensorflow 2.4.1, Pytorch 1.8.1, and OpenCV 4.1.2 were used as the libraries. open source uses YOLO_mark. Experimental procedure was as follows; 20 slides containing tubular basophilia and mineralization, which commonly occured spontaneous renal lesion in rats, were converted into WSI using a virtual slide scanner. After that, the training data were captured using the WSI reading program (Zen) and images containing each lesion were captured as jpg. After converting to a file, transfer learning was performed (also carried out on the jpg. files).

### Transfer learning of classification models

In the existing layer structures of InceptionV3, Xception, and MobileNetV2, the model was reconstructed in the order of the Dense (512) layer, the batch normalization layer, the Rectified Linear Unit (ReLU) activation function, the Dense (2) layer, and the softmax activation function. The batch size was 26, the optimizer was Adam. For the loss function, Categorical Cross Entropy was applied. After that, the model was trained after performing data pre-processing. Kidney lesions and normal tissues of various sizes were divided into 224 × 224 pixels per cell, and the images with smaller than in the 224 × 224 pixel area were fit into this area and supplemented with white background to adjust the size and then to adjust the size of the lesion. The image thus obtained was learned by classifying the recognized part and the unrecognized part.

### Transfer learning of the object detection model

The object detection model YOLOv4 was used, and the learning algorithm used to read kidney lesions proceeded in the following three steps: data bounding, data augmentation, and hyperparameter tuning. Data bounding used the YOLO_mark open source to bound the lesion to the training image. Considering that it is difficult to predict when boxes overlap, the boxes were bounded so that they did not overlap as much as possible. Mineralization was coded using Red Green Blue (RGB), and had a duller or darker colour than the surrounding tissue. In the case of tubular basophilia compared to normal tubules, coding was based on a dull colour, enlarged nucleus, and thickened basement membrane.

## Data Availability

All experiment data during this study are included in this manuscript.

## Abbreviations

AI: Artificial Intelligence
CPN: Chronic Progressive Nephropathy
CNN: Convolutional Neural Network
KNTP: Korea National Toxicology Program
mAP: Mean Average Precision
MFDS: Ministry of Food and Drug Safety
ReLU: Rectified Linear Unit
RGB: Red Green Blue
WSI: Whole Slide Image
YOLO: You Only Look Once
